# *PLOS Pathogens* 2015 Reviewer Thank You

**DOI:** 10.1371/journal.ppat.1005497

**Published:** 2016-02-23

The Public Library of Science (PLOS) and the *PLOS Pathogens* editorial team would like to express our tremendous gratitude to all individuals who participated in the peer review process for *PLOS Pathogens* this past year. As a community run journal, we depend on the participation of active and expert members of the field to communicate the best work. 2015 was great year for *PLOS Pathogens*; this year, 2,700 reviewers from around the world helped evaluate over 1,200 articles.

The names of our 2015 reviewers are listed in S1 Reviewer List. Thank you to all our reviewers for sharing your expertise with the *PLOS Pathogens* community. Your efforts are an important part of making the journal a highly read, highly cited and highly regarded open access resource created by and for the benefit of the scientific community.

## Supporting Information

S1 Reviewer List Alphabetical list of 2015 *PLOS Pathogens* reviewers. (PDF)
