# Supplementary material for: PLOS Pathogens 2015 Reviewer Thank You
Source: PLoS Pathog. 2016 Feb 23;12(2):e1005497. doi: 10.1371/journal.ppat.1005497 (PMC4764492; doi:10.1371/journal.ppat.1005497)
Supplement: S1 Reviewer List — (PDF) [file ppat.1005497.s001.pdf]

PLOS | PATHOGENS

PLOS Pathogens would like to thank all those who reviewed on behalf of the journal in 2015:

Derek Abbott
Allison Abendroth
Soman Abraham
David Abraham
Joana Abrantes
Gian Paolo Accotto
Mark Achtman
Margaret Ackerman
Hans Ackerman
Alvaro Acosta-Serrano
Toni Aebischer
Hervé Agaisse
Mavis Agbandje-Mckenna
Hector Aguilar
Patricia Aguilar
Sebastian Aguirre
Brian Ahmer
Jin-Hyun Ahn
Tero Ahola
Myles Akabas
Jan Albert
Randy Albrecht
John Alcorn
Anna Aldovini
Bree Aldridge
James Alexander
Mehrdad Alirezaei
Martin Allday
Lee-Ann Allen
Igor Almeida
Sam Alsford
J. Alspaugh
Nihal Altan-Bonnet
Galit Alter
Marcus Altfeld
Craig Altier
John Altman
Daniel Altmann
Neal Alto
Kishore Alugupalli
James Alwine
Ali Amara
Rama Rao Amara
Gaya Amarasinghe
Richard Ambinder
Zandrea Ambrose
Deborah Anderson
Tim Anderson
Deborah Anderson
Richard A Anderson
Brenna Anderson
Peter Anderson
David Andes
Christopher Andoniou
Olivier Andréoletti
Norma Andrews
Ourania Andrisani
Elliot Androphy
David Ann
Aftab Ansari
Nicholas Anstey
Cristian Apetrei
Heidi Appel
Judith Appleton
Hisashi Arase
Jacques Archambault
Nancie Archin
Ramon Arens
Pablo Argüeso
Glen Armstrong
Darius Armstrong-James
Michelle Arnold
David Aronoff
Cécile Arrieumerlou
Gustavo Arrizabalaga
Eurico Arruda
Janelle Arthur
Sassan Asgari
Aravind Asokan
Robert Atmar
Ina Attree
Walter Atwood
Pierre Aucouturier
Michelle Audsley
Victoria Auerbuch
Juan Aylion
Abdu Azad
Subash Babu
Steffen Backert
Taeok Bae
Fabio Bagnoli
Susanne Bailer
Justin Bailey
Anna Bakardjiev
Stephen Baker
Guus Bakkeren
Alejandro Balazs
Regina Baldini
Mitchell Balish
Jimmy Ballard
Niaz Banaei
Mark Banfield
Charles Bangham
James Bangs
Lawrence Banks
Xiaoyong Bao
Benoît Barbeau
Daniel L. Barber
Joseph Barbieri
Carolina Barillas-Mury
Edward Barker
Vincenzo Barnaba
Ellie Barnes
Thierry Baron
Ian Barr
Antonio Barragan
Simon Barratt-Boyes
Luke Barrett
Michael Barrett
Seth Barribeau
Peter Barry
Clifton Barry
Ralf Bartenschlager
Richard Bartfai
David Barton
Christopher Basler
Bonnie Bassler
Daniela Basso
Philippe Bastin

Thomas Baumert
Nicole Baumgarth
Andreas Baumler
Sina Bavari
Gillian Beamer
Michael Beard
Peter Beard
Bruno Beaumelle
James Beeson
Samuel Behar
Michael Behnke
Marcel Behr
Eduardo Bejarano
Gill Bejerano
Robert Belas
George Belov
Alexia Belperron
Graham Belsham
Robert Belshaw
Chris Benedict
Richard Bennett
Steven Bensinger
Leslie Berg
Howard Berg
Jeffrey Bergelson
Cornelia Bergmann
Vincent Beringue
Arnold Berk
Ben Berkhout
Kristen Bernard
Antonio Bertoletti
Brent Berwin
Gurdyal Besra
Sonja Best
Sonja Best
Sébastien Besteiro
Michael Betts
Stephen Beverley
Charles Bevins
Sumita Bhaduri-Mcintosh
Purnima Bhanot
Alok Bhattacharya
Martine Biard-Piechaczyk
Tihana Bicanic
Roman Biek
Ethan Bier
Elaine Bignell
R. Blake Billmyre
James Binley
William Bishai
Russell Bishop
Wilbert Bitter
Samuel Black
William Black Iv
David Blackbourn
Michael Blackman
Ira Blader
Andrew Blagborough
David Blair
Carol Blair
Nicolas Blanchard
Paola Blanchette
Steven Blanke
Jill Blankenship
Joel Blankson
David Blehert
James Bliska
Anna Blom
Jesse Bloom
David Bloom
Matthew Bochman
Linda Bockenstedt
Teun Boekhout
Debby Bogaert
Blaise Boles
Michael Bolker
Martino Bolognesi
David Boltz
Matteo Bonazzi
Ivo Boneca
Bryony Bonning
Marc Bonten
Henry Boom
Adrianus Boon
André Boonstra
Mike Boots
Kathleen Boris-Lawrie
Frederik Börnke
Monica Borucki
Ronald Bosch
Irene Bosch
Jeff Bose
Steven Bosinger
Fadila Bouamr
Yan Boucher
Steeve Boulant
Robert Bourret
Teun Bousema
Chris Boutell
Nicole Bouvier
Dawn Bowdish
Melissa Bowerman
Jon Boyle
Marcelo Bozza
Thomas Braciale
Doug Brackney
Peter Bradley
Kenneth Bradley
Curtis Brandt
Abraham Brass
Aaron Brault
Veronique Brault
Jonathan Braun
Catherine Braun-Breton
Alejandra Bravo
Jason Brenchley
Greg Brennan
Wade Bresnahan
Stephane Bressanelli
Volker Briken
Margo Brinton
Warwick Britton
Robert Britton
Mathieu Brochet
Nichole Broderick
Igor Brodsky
Barbara Broeker
Heike Broetz-Oesterhelt
David Brooks
Roland Brosch
Laurent Brossay
Eric Brown
Gordon Brown
Michael Brown
Jeremy Brown
Jay C Brown
Charles Brown
Dennis Brown
Jeffrey Browning
Petr Broz
Kenneth Bruce
Reinhold Brückner
John Brumell
Thijn Brummelkamp

Wolfram Brune
Patrick Brunner
Frédéric Brunner
Vincent Bruno
Emme Bruns
Clare Bryant
Mark Brynildsen
Juliane Bubeck Wardenburg
Nicolas Buchon
Carmen Buchrieser
Christopher Buck
Frederick Buckner
Marcus Buggert
Michael Bukrinsky
Marta Bull
Peter Bull
Lee Bulla
Dirk Bumann
Jozsef Burgyan
Barbara Burleigh
Wim Burmeister
Cara Burns
James Burns
Lori Burrows
Dennis Burton
Benjamin Burwitz
Michael Busch
Robin Bush
Noah Butler
Noah Butler
Ken Cadwell
Melissa Caimano
Richard Calendar
Guy Caljon
Caroline Cameron
Jennifer Cameron
Andrew Camilli
Gabriella Campadelli-Fiume
David Campbell
Edward Campbell
Yang Cao
Michael Caparon
Patrizia Caposio
Massimo Caputi
Rhonda Cardin
Andrea Carfi
Jonathan Carlson
George Carlson
Fredric Carlsson
Jason Carlyon
Elisabeth Carniel
John Carr
Vern Carruthers
Leonardo Carvalho
Josep Casadesús
Arturo Casadevall
Joseph Casazza
Eric Cascales
John Casey
Neil Cashman
James Cassanova
Maria Cassera
Clare Casteel
Joaquín Castilla
Maria Castillo
Maria Catanese
Robert Cattaneo
Giovanni Cattoli
Simon Cauchemez
Amy Caudy
Byron Caughey
Jean Celli
Adelheid Cerwenka
Ethel Cesarman
Marie-France Cesbron-Delauw
Ann Chahroudi
Wengang Chai
Debopam Chakrabarti
Edward Chan
Yoke Fun Chan
Eric Chan
Sumit Chanda
Bala Chandran
Kartik Chandran
Hsiao-Han Chang
Jeff Chang
Eugene Chang
Sylvain Charlat
Sujata Chaudhari
Ajay Chawla
Ian Cheeseman
Linda Chelico
Xiaojiang S Chen
Mei-Ru Chen
Benjamin Chen
Xiaojiang Chen
Fei Chen
Zheng Chen
Rong Chen
Steve Chen
Gong Cheng
Genhong Cheng
Cecilia Cheng-Mayer
Jacqueline Cherfils
Christophe Chevillard
Christine Chevillon
Rémi Cheynier
V. Gregory Chinchar
Nam-Hyuk Cho
Hyeryun Choe
Kyung Choi
Nicolas Chomont
Shan-Ho Chou
Claire Chougnet
Louise Chow
Neil Christensen
George Christophides
Konstantin Chumakov
Tae-Wook Chun
Michael Ciancanelli
Tomas Cihlar
Andrea Cimarelli
Christine Citti
Paul Clapham
François Clavel
Keith Clay
Christine Clayton
Rollie Clem
Janice Clements
David Clifford
Ellen Closs
Christine Clouser
Gitta Coaker
Cevayir Coban
Sarah Cobey
Alan Cochrane
Donald Coen
Jorn Coers
Lark Coffey
John Coffin
Kenneth Coggeshall
Taylor Cohen
Eric Cohen

Jeffrey Cohen
Randall Cohrs
David Colby
Caroline Colijn
Jérôme Collemare
Kathleen Collins
Marco Colonna
Tonya Colpitts
Richard Condit
David Conn
Elizabeth Connick
Nicholas Conrad
Klaus Conzelmann
Brian Coombes
Vaughn Cooper
Chester Cooper
Craig Coopersmith
Isabelle Coppens
Lawrence Corey
Brendan Cormack
Stephania Cormier
Omar Cornejo
Nicolas Corradi
Alfred Cortes
Mauro Cortez
Laurent Coscoy
Pascale Cossart
Francois-Loic Cosset
Barbara Coulson
Sarah Coulthurst
Kevin Couper
Mathieu Coureuil
Valerie Courgnaud
Timothy Cover
Leah Cowen
Benjamin Cowling
Carolyn Coyne
Alister Craig
Lisa Craig
Robert Craigie
Robert Cramer
Allan Cripps
Charles Criscione
Max Crispin
Alison Criss
Ileana Cristea
Daniel Croll
Peter Crompton
Nicholas Croucher
James Crowe, Jr.
Pal Csaba
Bryan Cullen
Valeria Culotta
James Culver
James Cunningham
Anthony Cunningham
Aubrey Cunnington
Stephen Curry
Stephen Cusack
Christopher Cutler
Jason Cyster
Björn Dahlbäck
Lisa Daley-Bauer
Tina Dalianis
Marc Dalod
Blossom Damania
Inger Damon
Inger Damon
Maura Dandri
Chi Dang
Jeffery Dangl
Jaiyanth Daniel
Pranav Danthi
Richard D'Aquila
Roy Dar
Richard Darveau
K.Heran Darwin
Saumitra Das
Atze Das
Gregory Dasch
Sana Dastgheyb
Sandip Datta
Matthew Daugherty
Miles Davenport
Robert Davey
Hume David
Jane Davies
Stephen Davies
Patricia Day
Raffaele De Francesco
Christopher De Graffenried
Ronnie De Jonge
Juan C. De La Torre
James Decaprio
Steven Deeks
George Deepe, Jr.
Victor Defilippis
Patrick Degnan
Rosa Maria Del Angel
Maurizio Del Poeta
Frank Deleo
Francis Delpeyroux
Neal Deluca
Barbara Demmig-Adams
Christophe D'Enfert
Hongyu Deng
Qing Deng
Mark Denison
Eric Denkers
David Dennings
Alexander Dent
Cynthia Derdeyn
Vojo Deretic
Terence Dermody
Isabelle Derré
Jeremy Derrick
Prashant Desai
Albert Descoteaux
Ronald Desrosiers
Scott Dessain
Ulrich Desselberger
Johannes Dessens
Anne Marie Di Guilmi
Michael Diamond
Arturo Diaz
Maureen Diaz
Felipe Diaz-Griffero
Martin Dickman
Andreas Diefenbach
Binh Diep
Ralf Dietzgen
Bernhard Dietzschold
Stephanie Diezmann
Paul Digard
Ivan Dikic
Adler Dillman
Daniel Dimaio
Nigel Dimmock
George Dimopoulos
Maria D'Império Lima
Rhoel Dinglasan
Ron Diskin
Meike Dittmann
Dirk Dittmer

Maziar Divangahi
Richard Dix
Linda Dixon
Armin Djamei
Julianne Djordjevic
Thomas Dobner
David Dockrell
Peter Dodds
Tamara Doering
Gilad Doitsh
Terje Dokland
Robert Doms
Min Dong
Xinnian Dong
Suomeng Dong
Anna Dongari-Bagtzoglou
Ruben Donis
Michael Donnenberg
John Doorbar
Katie Doores
Martin Dorf
Margherita Doria
Pedro D'Orleans-Juste
Charles Dorman
Katerina Dorovini-Zis
Daniel Douek
Janet Douglas
John Dow
John Downie
Rika Draenert
Shaynoor Dramsi
Dan Drecktrah
Christian Drosten
Peter Dube
David Dubnau
Jean Dubuisson
Erin Dueber
Patrick Duffy
Michael Duffy
Roy Duncan
Sarah Dunstan
Sébastien Duplessis
W Paul Duprex
Joan Durbin
Michael Duszenko
Rebecca Dutch
Jonathan Dworkin
Kelly Dyer
Roman Dziarski
Daniel Ebbole
Gregory Ebel
Matthias Eberl
Leo Eberl
Hideki Ebihara
Lars Eckmann
Paul Edlefsen
W. John Edmunds
Stacey Efstathiou
Bernhard Ehlers
Roselyn Eisenberg
David Eisenberg
Laurence Eisenlohr
Wolfgang Eisenreich
Amie Eisfeld
Nels Elde
Gillian Elliott
Candace Elliott
Jerrold Ellner
Mohamad El-Zaatari
Vince Emery
Richard Enelow
Joanne Engel
Alan Engelman
David Engman
Christian Engwerda
Jost Enninga
Lynn Enquist
Laura Ensign
Alexander Ensminger
Armin Ensser
Marc Erhardt
Ingemar Ernberg
Joel Ernst
Robert Ernst
Joseph Eron
José Esté
Jacob Estes
Mary K. Estes
Pedro Esteves
Agustin Estrada-Pe±A
Judy Estroff
Thomas Eulgem
David Evans
David Evans
Tom Evans
Matthew Evans
David Evans
Roger Everett
Jonathan Ewbank
Oliver Fackler
Abeer Fadda
Alan Fairlamb
Erik Falck-Pedersen
Bryce Falk
Ferric Fang
Ying Fang
Donna Farber
Paul J Farrell
Antonio Fasanella
Herman Favoreel
Michael Federle
Barbara Felber
Mario Feldman
Heinz Feldmann
Marta Feldmesser
Jacques Fellay
Pinghui Feng
Zongdi Feng
Michael Ferguson
Sara Ferrando-Martinez
Dominique Ferrandon
Julia Ferrari
Richard Ferrero
Martin Ferris
Mark Field
Melania Figueroa
Monika Fijak
Sergio Filipe
Scott Filler
Alain Filloux
Robert Finberg
Keisha Findley
Peter Fineran
Katja Fink
Stefan Finke
Natosha Finley
Andrew Firth
Nicole Fischer
Vincent Fischetti
Matthew Fisher
Mark Fisher
Katherine Fitzgerald
Marie Flamand
Katie Flanagan

Richard Flavell
James Fleckenstein
Suzanne Fleiszig
John Fleming
Erik Flemington
Michelle Flenniken
S. Flint
Lucile Floeter-Winter
Laurence Florens
Ervin Fodor
Beatriz Fontoura
Chris Ford
Katrina Forest
J. Forrest
Sarah Fortune
Timothy Foster
Simon Foster
Ron Fouchier
Vance Fowler
Ellen Fox
Nicole Frahm
Olivera Francetic
Genoveffa Franchini
Dara Frank
Gad Frankel
Bernardo Franklin
Christophe Fraser
John Fraser
James Fraser
Nigel Fraser
Claire Fraser
John Frater
Eric Freed
Nancy Freitag
Jeffrey Frelinger
Lisa Frenkel
Ute Frevert
Matthew Frieman
Timothy Friesen
Friedrich Frischknecht
Ilya Frolov
Yangxin Fu
Yukako Fujinaga
Takashi Fujita
Benjamin Fulton
Clay Fuqua
Dahlene Fusco
Gülsah Gabriel
Dana Gabuzda
Michaela Gack
Jennifer Gaddy
Sarah Gaffen
Sebastien Gagneux
Michael Gale Jr.
Thomas Gallagher
Teresa Gallart
Benoît Gamain
Andrea Gamarnik
Yunn-Hwen Gan
Thumballi Ganapathi
Roman Ganta
Feng Gao
Qian Gao
David Garboczi
Robert Garcea
Juan Antonio García
Hernan Garcia-Ruiz
Adolfo Garcia-Sastre
Donald Gardiner
Melanie Gareau
Nisha Garg
Danielle Garsin
Pablo Gastaminza
Yves Gaudin
Elodie Gaulin
William Gause
Gregory Gauthier
Ricardo Gazzinelli
Timothy Geary
Adam Geballe
Adam Gehring
Teunis Geijtenbeek
Caroline Genco
Giuilo Genovese
Thomas German
James Gern
Elodie Ghedin
Frank Gherardini
Melanie Ghoul
Mauro Giacca
Paul Giacomin
Chou-Zen Giam
Wade Gibson
Robert Gifford
Mark Gijzen
Tim Gilberger
Clement Gilbert
John Gilleard
Sarah Gilmore
Ole Gjoerup
Britt Glaunsinger
Jennifer Gleason
Anthony Glenn
Rudolph Glockshuber
Dale Godfrey
Marielle Gold
Joanna Goldberg
Daniel Goldberg
William Goldman
Kenneth Gollob
Mark Gomelsky
Marisa Gómez
Mercedes Gonzalez-Juarrero
Michael Goodin
Felicia Goodrum
Nilu Goonetilleke
Alexander Gorbalenya
Christian Gortazar
Heinrich Gottlinger
Patrice Gouet
Caroline Goujon
Keith Gould
Mark Goulian
Richard Gourse
Neil Gow
Yonatan Grad
David Grainger
John Grainger
Arash Grakoui
Thomas Gramberg
Donald Granger
Nicholas Grassly
Johannes Graumann
Frederik Graw
Valery Grdzelishvili
Mel Greaves
Urs Greber
Kim Green
Sharone Green
Patrick Green
William Green
Patrick Green
Todd J Green
Warner Greene

Richard Grencis
Finn Grey
Nicole Grieshaber
Gareth Griffith
Anthony Griffiths
Michael Grigg
Tamara Gritsun
Jane Grogan
Eduardo Groisman
Chuck Grose
Allison Groseth
Christina Grozinger
Angelika Grundling
Christoph Grundner
Niklaus Grunwald
Yi Guan
John Guatelli
Marc-Jan Gubbels
Andrei Gudkov
Pierre Guermonprez
Christophe Guilhot
Karen Guillemin
Nancy Guillen
Susana Guix
Erich Gulbins
Paul Gulig
Keith Gull
Suryaram Gummuluru
John Gunn
Feng Guo
Haitao Guo
Nishith Gupta
Pawan Gupta
Andrea Gust
Ana Lorena Gutiérrez Escolano
David Guttman
Bart Haagmans
Rainer Haas
Georg Häcker
Elias Haddad
Julius Clemence Hafalla
Nancy Haigwood
Stephen Hajduk
George Hajishengallis
William Halford
Elissa Hallem
Dirk Haller
Patrick Hamilton
Sven Hammerschmidt
Wolfgang Hammerschmidt
Katie Hampson
Jiahuai Han
Meaghan Hancock
Lynn Hancock
Dorit Hanein
Willem Hanekom
Lars Hangartner
Kathryn Hanley
Linda Hanley-Bowdoin
Philip Hanna
Diana Hansen
Ashraful Haque
Wolf-Dietrich Hardt
Philip Hardwidge
Richard Hardy
Edward Harhaj
Nicola Harris
Reuben Harris
Steven Harris
Mark Harris
James Harris
Tom Harrison
Stephen Harrison
Rasika Harshey
Dominik Hartl
Elizabeth Hartland
Susanne Hartmann
Ronald Harty
John Harty
Daniel Hassett
Masanori Hatakeyama
Christof Hauck
Norman Haughey
Florencia Haurat
Vasili Hauryliuk
Alan Hauser
John Hawdon
Thomas Hawn
Chuan He
Xiaosong He
Patrick Hearing
Aoife Heaslip
Nicholas Heaton
David Heckel
Stephen Hedrick
Ralf Heermann
Antje Heese
Richard Hegele
Johannes Hegemann
Adrian Hehl
Bernd Heimrich
Franz Heinz
Robert Heinzen
Mark Heise
Sophie Helaine
Ekaterina Heldwein
Christopher Hellen
Helena Helmby
Els Henckaerts
David Hendrixson
Timothy Henrich
Thomas Henry
Michael Hensel
Lisa Hensley
Scott Hensley
Georges Herbein
Andrew Herbert
Debroski Herbert
Raquel Hernandez
Susanne Herold
Andrew Herr
Johannes Herrmann
Anat Herskovits
Tomer Hertz
Alejandro Heuck
Volker Heussler
James Hewitson
Matthew Higgins
Stephen Higgs
Hubert Hilbi
William Hildebrand
Rolf Hilgenfeld
Ann Hill
Andrew Hill
Julian Hillyer
B. Joseph Hinnebusch
Hans Hirsch
Alec Hirsch
Robert Hirt
John Hiscott
Amy Hise
Andrew Hislop
Ling-Pei Ho

Lucas Hoffman
Monica Höfte
Deborah Hogan
James Hogle
Kristin Hogquist
Brenda Hogue
Tobias Hohl
Michael Holbrook
David Holden
Lindy Holden-Dye
Steven Holland
Edward Holmes
Fred Homa
Masao Honda
Michael Hood
Magnus Hook
Carol Hopkins Sibley
David Horn
Mathias Hornef
Stacy Horner
Mady Hornig
Veit Hornung
Malcolm Horsburgh
Alexander Horswill
Curt Horvath
Jonathan Howard
James Hoxie
Michael Hsieh
Jianming Hu
Xiaoyu Hu
Kerwyn Huang
Yan-Jang Huang
Bernhard Hube
Sally Huber
Stephen Hughes
David Hughes
Grant Hughes
Stephen Hughes
Edgar Huitema
Scot Hulbert
Scott Hultgren
Dan Hultmark
Judith Humphries
David Hunstad
Peter Hunt
Eric Hunter
Christopher Hunter
Jason Huntley
Greg Hurst
Christopher Huston
Edward Hutchinson
William Hutchison
Lindsey Hutt-Fletcher
Lars Hviid
Seungmin Hwang
Matteo Iannacone
Alexander Idnurm
James Imlay
Jean-Luc Imler
Michael Imperiale
Robin Ingalls
Kyung-Soo Inn
Roger Innes
Naohiro Inohara
Ronald Iorio
Ralph Isberg
Antonella Isgro
Ken Ishii
Masayuki Ishikawa
Wannaporn Ittiprasert
Ivaylo Ivanov
Dmitri Ivanov
Luis Izquierdo
Yoshihiro Izumiya
Mary Ann Jabra-Rizk
Andrew Jackson
William Jackson
James Jacobberger
Thomas Jacobs
Marc Jacobsen
Leo James
Julie Jameson
Amanda Jamieson
James Jancovich
Dragana Jankovic
David Jans
Edith Janssen
Ronald Javier
Stephen Jenkins
Michael Jennings
Grant Jensen
Keith R Jerome
Holger Jeske
Zhengfan Jiang
Xia Jin
Dong-Yan Jin
Rongsheng Jin
Eric Johannsen
Cecilia Johansson
Mina John
Pål Johnsen
Christine Johnson
Jack Johnson
Patricia Johnson
David Johnson
Karyn Johnson
Marc Johnson
Reed Johnson
Welkin Johnson
R. Paul Johnson
Kay Johswich
Clinton Jones
Ian Jones
Clinton Jones
Jonathan Jones
Frans Jongejan
Stipan Jonjic
Wilfried Jonkers
Colleen Jonsson
Michael Jordan
Martha Jordan
Joyce Jose
Christine Josenhans
Sarah Joseph
Kirsten Jung
Wayne Jurick
Edouard Jurkevitch
Sheryl Justice
Sheryl Justice
Aras Kadioglu
David Kadosh
Susan Kaech
Jonathan Kagan
Kevin Kain
Markus Kainulainen
William Kaiser
Spyros Kalams
Shiv Kale
Robert Kalejta
Axel Kallies
Satya Kalluri
Daniel Kalman
Isgouhi Kaloshian
Martin Kaltenpoth

Jeremy Kamil
Sophien Kamoun
Bavesh Kana
Seogchan Kang
Michael Kann
Mari Kannagi
Geetha Kannan
Thirumala-Devi Kanneganti
Daniel Kaplan
Stefan Kappe
Jonathan Karn
Timothy Karr
Stephanie Karst
Gunasegaran Karupiah
Fatah Kashanchi
Takahito Kashiwagi
Peter Kasson
Sudhir Kasturi
Christine Katlama
Susumu Katsuma
Michael Katze
Paul Kaufman
Daniel Kaufmann
Amitinder Kaur
Deepak Kaushal
Charu Kaushic
Annemieke Kavelaars
Ikuro Kawagishi
Yasushi Kawaguchi
Thomas Kawula
Joseph Keane
Nancy Kedersha
Dean Kedes
Katherine Kedzierska
Brandon Keele
Patrick Keeling
Thomas Kehl-Fie
Anthony Kelleher
Thomas Kelly
David Kelly
John Kelly
Brian Kelsall
Volkhard Kempf
Bettina Kempkes
Melissa Kendall
Shannon Kenney
Scott Kenney
Linda Kenney
Brendan Kenny
Oliver Keppler
Mehmet Kesimer
Olen Kew
Vineet Kewalramani
Shabaana Khader
Imtiaz Khan
Chang Hyun Khang
Kamal Khanna
Sangeeta Khare
Alexander Khromykh
Margaret Kielian
Tammy Kielian
Baek Kim
Kami Kim
Peter Kima
Paul Kinchington
Robert Kingsley
Frank Kirchhoff
Uday Kishore
Scott Kitchen
Tohru Kiyono
Per Johan Klasse
Nichole Klatt
Bruce Klein
Michael Klemba
Kimberly Kline
Aloysius Klingelhutz
Karl Klose
Dagmar Knebel-Mörsdorf
Rob Knight
Matty Knight
David Knipe
Leigh Knodler
Dennis Ko
Tetsuro Kobayashi
Scott Kobayashi
Atsushi Kobayashi
Lester Kobzik
Julia Koehler
Andrew Koh
Alain Kohl
Linda Kohl
Tamar Kohn
Michael Kolbe
Jay Kolls
Kouacou Konan
James Konopka
Michael Koomey
Manfred Kopf
Bette Korber
Hardy Kornfeld
Daniel Kornitzer
Anita Koshy
Sergei Kotenko
Susan Koval
Pavel Kovarik
Susan Kovats
Thomas Kozel
Pamela Kozlowski
Florian Krammer
Duncan Krause
Eric Kremer
Manoj Krishnan
Thomas Kristie
Bastiaan Krom
James Kronstad
Pascale Kropf
Laurie Krug
Eric Krukonis
Damian Krysan
Urszula Krzych
Paul Kubes
Adam Kucharski
Karl Kuchler
Meta Kuehn
Ralf Kueppers
Jens Kuhn
Richard Kuhn
Thijs Kuiken
Jon Kull
Yutaro Kumagai
Purnima Kumar
Gael Kurath
Joachim Kurtz
Jorgen Kurtzhals
Sergei Kusmartsev
Mamuka Kvaratskhelia
Douglas Kwon
Nicole La Gruta
Celia Labranche
Nadine Laguette
Michael Lagunoff
Erh-Min Lai
Lou Laimins
Anna-Liisa Laine

Seema Lakdawala
Jean-François Laliberté
Kong-Peng Lam
Robert Lamb
Tracey Lamb
Olivier Lambotte
David Lambright
Richard Lamont
Ke Lan
Nathaniel Landau
Alan Landay
Scott Landfear
Santo Landolfo
Thomas Lane
Ryan Langlois
Gordon Langsley
Lewis Lanier
Joseli Lannes-Vieira
Fanny Lanternier
Antonio Lanzavecchia
Göran Larson
Iñigo Lasa
Jean-Paul Latge
Wyndham Lathem
Chris Lauber
Georg Lauer
Adam Lauring
Grégoire Lauvau
Thomas Lavstsen
Mansun Law
Rachel Lawrence
Brian Lazzaro
Tiziana Lazzarotto
Karine Le Roch
Brian Leander
Alice Lebreton
Marc Lecuit
Michael Lederman
Chia Lee
Won-Jae Lee
Sujin Lee
Bok-Luel Lee
Yin-Won Lee
Robyn Lee
Kevin Legge
Giuseppe Legname
Adele Lehane
Paul Lehner
David Leib
Salome Leibundgut-Landmann
Giorgio Leigheb
Petr Leiman
Bruno Lemaitre
Isabelle Lemasson
Philippe Lemey
Niels Lemmermann
Deborah Lenschow
Laurel Lenz
Jean Lepault
Julien Lescar
Scott Letendre
Anthony Leung
Elena Levashina
Andrew Lever
Jose Levi
Petra Levin
Randall Levings
Stuart Levitz
David N Levy
Sharon Lewin
Amanda Lewis
George Lewis
Jennifer Lewis
Kui Li
Jianrong Li
Jonathan Li
Guangpu Li
Wenhui Li
Yi Li
Xin Li
Xiao-Dong Li
Fang Li
Ziyin Li
Ming Li
Chen Liang
Chengyu Liang
T. Jake Liang
Mathias Lichterfeld
Paul Lieberman
Egil Lien
Jan Liese
Jeffrey Lifson
Hauke Lilie
Christopher D. Lima
Philana Lin
Zhen Lin
Na-Sheng Lin
Wenyu Lin
Gunnar Lindahl
Brett Lindenbach
Paul Ling
Michail Lionakis
John Lipuma
Tom Little
Tom Little
Shan-Lu Liu
Yancheng Liu
Cindy Liu
George Liu
Sijun Liu
Yule Liu
Manuel Llinás
Richard Lloyd
James Lloyd-Smith
Melissa Lodoen
Daniel Loeb
Bettina Loeffler
Volker Lohmann
Volker Lohmann
Shee Mei Lok
P'Ng Loke
Richard Lo-Man
George Lomonossoff
David Long
Richard Longnecker
Ulisses Lopes
Carolina Lopez
Miguel López-Botet
Juan Jose Lopez-Moya
Karin Lore
Michael Lorenz
Zdravko Lorkovic
Jennifer Loros
Stephen Lory
Joe Louis
Alex Loukas
Sebastian Lourido
Philip Loverde
Anice Lowen
Tao Lu
Jeremy Luban
Paul Luciw
Shirley Luckhart

Stephan Ludwig
Micah Luftig
Nicholas Lukacs
Gary Luker
Julius Lukes
Jennifer Lund
Patric Lundberg
Zhao-Qing Luo
Ming Luo
Honglin Luo
Marina Lusic
Priya Luthra
Katherine Luzuriaga
Hinh Ly
Gareth Lycett
Rebecca Lynch
Zhonghua Ma
Stuart Macdonald
Michael Mach
Matthias Machner
Jason Mackenzie
David Mackey
James MacLachlan
Annette MacLeod
Hiten Madhani
Stefan Magez
Melissa Maginnis
Gkikas Magiorkinis
Diogo Magnani
Siddhartha Mahanty
Renaud Mahieux
Rick Maizels
Kristiina Mäkinen
Subramaniam Malarkannan
Frank Maldarelli
Ray Malfavon-Borja
Richard Malley
Kalle Malmberg
Mark Mandel
Ofer Mandelboim
Judith Mandl
Nicolas Manel
Nicholas Maness
Evelyne Manet
Adhar Manna
John Mansfield
Jean Manson
Anil Mantha
Ivan Marazzi
Richard Marconi
Joseph Marcotrigiano
František Marec
Leonid Margolis
Todd Margolis
David Margolis
Chelsea Marie
Kevin Maringer
Juan Marini
Wilfred Marissen
Joao Marques
Aron Marquitz
Kevin Marsh
An Martel
Matthias Marti
Gregory Martin
Roland Martin
Richard Martin
Javier Martin
Miguel Martin-Acebes
Adrian Martineau
Elena Martinelli
Esteban Martinez
Juan Martinez
Luis Martínez-Sobrido
Nelson Martins
Preston Marx
Amanda Marzo
John Mascola
Pascal Mäser
Vega Masignani
Pietro Mastroeni
Maria Masucci
Robin Mathew
Jyl Matson
Masao Matsuoka
Yoshiharu Matsuura
Kai Matuschewski
Wendy Maury
Robin May
Katrin Mayer-Barber
Charles Mays
Sarkis Mazmanian
Alison McBride
Shonna McBride
Dennis McCance
Douglas McCarty
Bruce McClane
Malcolm McConville
A. Louise McCormick
Craig McCormick
Joseph McCune
Christopher McDevitt
Sarah McDonald
Julie McDonald
John McDowell
Anita McElroy
Alastair McEwan
Grant McFadden
Johnjoe McFadden
Maureen McGargill
Dorian McGavern
Lesley McGee
James McGettigan
Elizabeth McGraw
Kevin McIver
John McLauchlan
Margaret McLaughlin-Drubin
Jason McLellan
Diane McMahon-Pratt
W. Robert McMaster
Andrew McMichael
Finlay McNab
Helen McShane
Stephen McSorley
Khisimuzi Mdluli
Joan Mecsas
Eva Medina
Andrew Mehle
Jeffery Meier
Jacques Meis
Peter Melby
Ulrich Melcher
Thomas Melendy
Jose Melero
John Mellors
Robert Menard
Patricio Meneses
Tesfaye Mengiste
Jason Mercer
Juanita Merchant
Seppo Meri
Houra Merrikh
Janet Mertz
Ilhem Messaoudi

William Messer
Martin Messerle
Thomas Mettenleiter
Dennis Metzger
Thomas Meyer
Austin Meyer
Blake Meyers
Craig Meyers
Edward Miao
Shulamit Michaeli
Ivan Mijakovic
W. Allen Miller
Matthew Miller
Samuel Miller
George Miller
William Miller
Jeff Miller
Jeffrey Miller
Louis Miller
Wolfgang Miller
Danny Milner, Jr.
Booki Min
Olivo Miotto
Chad Mire
Dominique Missiakas
Aaron Mitchell
Edward Mitre
Guillaume Mitta
Makoto Miyata
Joseph Mizgerd
Valerie Mizrahi
Mahtab Moayeri
Harry Mobley
Edward Mocarski
Yorgo Modis
Robert Modlin
Ian Mohr
Susan Moir
Fumitaka Momose
Denise Monack
Mario Mondelli
Luis Montaner
David Montefiori
Susan Montgomery
Cary Moody
Branch Moody
M. Moody
Margo Moore
Bethany Moore
John Moore
Penny Moore
Jonathan Moorman
Ann Moormann
Andreas Moosmann
Darius Moradpour
Thomas Moran
Silvia Moreno
Renato Morona
James Morris
Lynn Morris
Lynda Morrison
Thomas Morrison
Donald Morrison
Liam Morrison
Naomi Morrissette
Nathan Mortimer
Anne Moscona
Donald Mosier
Bernard Moss
David Mosser
Karen Mossman
Serge Mostowy
Maria Mota
Md Motaleb
Walther Mothes
Vladimir Motin
Jeremy Mottram
Scott Moye-Rowley
Samira Mubareka
Mary Beth Mudgett
Scott Mueller
Shaeri Mukherjee
Amitabha Mukhopadhyay
Harshini Mukundan
Marcel Müller
Michaela Müller-Trutwin
Matthew Mulvey
Ulrike Munderloh
Joshua Munger
Sandie Munier
Maite Muniesa
Carsten Munk
Carol Munro
Vincent Munster
Christian Munz
Pablo Murcia
Eain Murphy
Paul Murray
Peter Murray
Jeff Murry
Nick Muzyczka
Peter Myler
Joe Mymryk
Gary Nabel
Nadia Naffakh
Hiroki Nagai
Uma Nagarajan
Peter Nagy
Meera Nair
Savita Nair
Venugopal Nair
Rajesh Nair
Koji Nakayama
Jarlath Nally
Franz Narberhaus
David Narum
Michael Nassal
Jim Nataro
William Nauseef
Hans Nauwynck
Francis Ndungu
Thumbi Ndung'U
Melody Neely
Matthew Neiditch
Stuart Neil
Andrew Neish
Martha Nelson
Glen Nemerow
Mihai Netea
Gabriele Neumann
Michael Nevels
Mari-Anne Newman
Hayley Newton
Olivier Neyrolles
Dao Nguyen
Tracy Nicholson
Kirsten Nielsen
Stefan Niemann
Michael Niepmann
Amelia Nieto
Jérôme Nigou
Fangkun Ning
Victor Nizet
Hiroaki Noda

Derek Nolan
Karen Norris
Steven Norris
Mairi Noverr
Tomoyoshi Nozaki
Evgeny Nudler
Jack Nunberg
Donald Nuss
Susanne Nylen
Anne O’Garra
Joshua Obar
Joshua Obar
Torsten Ochsenreiter
Roberta O'Connor
Christine O'Connor
Una O'Doherty
Julia Oh
Peter O'Hare
Päivi Ojala
Hiroaki Okamoto
Ana Oleaga
Andrew Olive
Martin Olivier
Vesa Olkkonen
Matthew Olson
Michal Olszewski
James Omichinski
Akira Ono
Steven Opal
Ariella Oppenheim
Carlos Orihuela
Mary O'Riordan
Amos Orlofsky
David Ornelles
Kim Orth
Andrew Osborne
Campetella Oscar
Nir Osherov
Hiroyuki Oshiumi
Nikolaus Osterrieder
Mary O'Sullivan
Melanie Ott
Tom Ottenhoff
Michael Otto
Julie Overbaugh
Annette Oxenius
Michelle Ozbun
Antonio Pagan
Savita Pahwa
Anand Pai
Mirko Paiardini
Peter Palese
Massimo Palmarini
Ann Palmenberg
Brent Palmer
Søren Paludan
Qinghua Pan
Quintin Pan
Gianfranco Pancino
John Panepinto
Giuseppe Pantaleo
Ralph Pantophlet
Krisztina Papp-Wallace
Catherine Paradis-Bleau
Leslie Parent
Colin Parrish
Chris Parsons
Marcela Pasetti
Diana Pastrana
James Paton
John Patton
Eva-Katharina Pauli
James Paulson
Silke Paust
Martin Pavelka
George Pavlakis
William Paxton
Robert Paxton
Eric Pearlman
Melanie Pearson
Scott Peck
Joao Pedra
R Peebles, Jr.
Richard Peek
Mark Peeples
Olve Peersen
Raymond Peirce
J.S. Malik Peiris
Andrew Pekosz
Vladimir Pelicic
Philip Pellet
Jose Penades
Carlos Penha-Goncalves
Francois Penin
Marta Perego
Lenore Pereira
Alan Perelson
Rushika Perera
Daniel Perez
Steven Perlman
Stanley Perlman
Sallie Permar
Matthieu Perreau
Deborah Persaud
Katarina Persson
Patricia Pesavento
Andreas Peschel
Nathan Peters
Brian Peters
Benjamin Petre
William Petri, Jr.
Virginie Petrilli
Constantinos Petrovas
Melinda Pettigrew
Sebastien Pfeffer
Julie Pfeiffer
Herbert Pfister
Jennifer Philips
Margaret Phillips
Caroline Philpott
Andreas Pichlmair
Raymond Pickles
Gerald Pier
Susan Pierce
Philippe Pierre
Theodore Pierson
Thomas Pietschmann
Thomas Pietschmann
David Pigott
Vincent Piguet
Satish Pillai
Mariana Pinho
Jaume Pinol
David Pintel
Claudine Pique
Hanspeter Pircher
Cynthia Pise-Masison
Barry Pittendrigh
Bodo Plachter
Paul Planet
Richard Plemper
Christopher Plowe
Kim Plummer
Maurizio Pocchiari

Eric Poeschla
Judit Pogany
Joe Pogliano
Stefan Pöhlmann
Stefan Pöhlmann
Pascal Poignard
Alexander Poltorak
Stephen Polyak
Carolina Poncini
Mikhail Pooggin
Art Poon
Michel Popoff
Owen Pornillos
Matteo Porotto
Thibaud Porphyre
Silvia Portugal
Mary Poss
Carol Post
Michael Potchen
Robert Poulin
Michael Povelones
Ann Powers
Gabriele Pradel
Daniel Prantner
James Prestegard
Peter Prevelige
Chester Price
Judith Prieto
Alice Prince
Martin Prlic
Steven Projan
Ulrike Protzer
Jose Puente
Stefan Pukatzki
Damian Purcell
Michael Purdy
Germain Puzo
Dohun Pyeon
Qiwei Qin
Xiangguo Qiu
Xiayang Qiu
Jin-Long Qiu
Feng Qu
Luis Quadri
Janet Quinn
Kylie Quinn
Francisco Quintana
Gabriel Rabinovich
Vincent Racaniello
Justin Radolf
Sheli Radoshitzky
Magdalena Radwanska
Manuela Raffatellu
Abdul Hakkim
Rahamathullah
Laurence Rahme
Tracy Raivio
Lawrence Rajendran
Glenn Rall
Katherine Ralston
Sanjay Ram
Gordon Ramage
Kumaran Ramamurthi
Sasirekha Ramani
Mika Rämet
Kyle Ramsey
Richard Randall
Glenn Randall
Felix Randow
Lisa Ranford-Cartwright
Zihe Rao
Didier Raoult
Jayne Raper
Chad Rappleye
Jason Rasgon
Michael Ratcliffe
Poonam Rath
Phil Rather
John Rathjen
Adam Ratner
Lee Ratner
David Raulet
Sebastian Rausch
Pierre-Emmanuel Rautou
Ranjit Ray
Julian Rayner
Brandon Razooky
Elizabeth Read
Laurie Read
Leslie Real
Walt Ream
Andrew Redd
Alec Redwood
Douglas Rees
R. Keith Reeves
Roland Regoes
Barbara Rehermann
Nancy Reich
Jean-Marc Reichhart
Steven Reiner
Celso Reis
William Reisen
David Rekosh
Alan Remaley
Han Remaut
Jyothi Rengarajan
Laurent Rénia
Rolf Renne
Martijn Rep
Jesus Requena
Félix Rey
Hugh Reyburn
Arturo Reyes-Sandoval
Stuart Reynolds
Todd Reynolds
Ruy Ribeiro
Andrew Rice
Charles Rice
Peter Rice
Stephen Rice
Thomas Richards
Christopher Richardson
Anthony Richardson
Douglas Richman
Rebecca Rico-Hesse
Thomas Ried
Arne Rietsch
Howard Riezman
Steven Riley
Sylvie Rimsky
Charles Rinaldo
Rita Rio
Amariliz Rivera
Frazer Rixon
Merlin Robb
Marjorie Robert-Guroff
Sigrid Roberts
Erle Robertson
Richard Robinson
Jean-Christophe Rochet
Rosemary Rochford
Tonie Rocke
Daniel Rockey
Isabel Roditi

Mauricio Rodrigues
Ana Rodriguez
Andrew Roe
Ute Roemling
Thierry Roger
David Rogers
Stephen Rogerson
Michael Roggendorf
John Rohde
Holger Rohde
George Rohrmann
Antonis Rokas
Richard Roller
Jeffrey Rollins
Patricia Romano
Christophe Romier
Suzan Rooijakkers
Cliona Rooney
Roy Roop Ii
Ray Roos
Pierre Roques
Patricia Rosa
Adriana Rosato
John Rose
Ilan Rosenshine
Peter Rosenthal
Joshua Ross
Ornelle Rossetto
Michael Rossmann
Stefan Rothenburg
Simon Rothenfusser
Alex Rowe
Dean Rowe-Magnus
David Rowlands
Syamal Roy
Chad Roy
Craig Roy
Thomas Rudel
Gloria Rudenko
Lucien Rufener
Natividad Ruiz
H. Earl Ruley
Steffen Rupp
David Russell
Eugenia Russinova
Zsolt Ruzsics
Zsolt Ruzsics
Robert Ryan
Wang-Shick Ryu
Julie Saba
Sead Sabanadzovic
Suraj Sable
Jonah Sacha
David B. Sacks
David L. Sacks
Subash Sad
Ivan Sadowski
Jeroen Saeij
Asier Sáez-Cirión
Saveez Saffarian
Selena Sagan
Manish Sagar
Yusuke Saijo
Takeshi Saito
Takemasa Sakaguchi
Suehiro Sakaguchi
Juan Salazar
Maria Carla Saleh
Maxim Salganik
Anna Salvetti
Suryaprakash Sambhara
Venkatesh Sampath
Clare Sample
Michel Samson
Charles Samuel
John Samuelson
R. Samulski
Andrea Sanchez-Vallet
Matyas Sandor
Rozanne Sandri-Goldin
Hélène Sanfaçon
Dominique Sanglard
Krishna Sanjeev
Andrea Sant
Martin Sapp
Antonio Sarikas
Saumendra Sarkar
Peter Sarkies
Peter Sarnow
Christopher Sassetti
Zsuzsanna Sasvari
Karla Satchell
Quentin Sattentau
Karin Sauer
John-Demian Sauer
Robert Sauerwein
Ram Savan
Tor Savidge
Dorothea Sawicki
Nancy Sawtell
Sara Sawyer
Timothy Schacker
Brian Schaffhausen
Luis Schang
Hermann Schätzl
Mark Schembri
Jeffrey Schertzer
Richard Scheuermann
Giampietro Schiavo
William Schief
Dieter Schifferli
John Schiller
Richard Schlegel
Mark Schleiss
Larry Schlesinger
Patrick Schlievert
Dirk Schluter
Michael Schmid
Anthony Schmitt
Gerold Schmitt-Ulms
Jörn Schmitz
Olaf Schneewind
Tanja Schneider
David Schneider
Matthias Schnell
Christoph Schoen
Stephen Schoenberger
John Schoggins
Gabriele Schönian
Tony Schountz
Gideon Schreiber
Kate Schroder
Alexandra Schubert-Unkmeir
Stacey Schultz-Cherry
Thomas Schulz
Walter Schulz-Schaeffer
Stephan Schwander
Ira Schwartz
Olivier Schwartz
Frank Schweizer
Heinzpeter Schwermer
Benjamin Schwessinger
Phillip Scott
Rona Scott

Gavin Screaton
Thomas Scriba
Eileen Scully
Michael Seaman
Matthew Seaman
Kimberley Seed
Christoph Seeger
Michael Seidl
Liisa Selin
James R. Sellers
Ganes Sen
Adrish Sen
Irini Sereti
Ruth Serra-Moreno
Peter Setlow
Alessandro Sette
Stephanie Seveau
David Severson
Idit Shachar
William Shafer
Yechiel Shai
Libo Shan
Michal Shapira
Neelam Sharma-Walia
Amir Sharon
Megan Shaw
Albert Shaw
Samuel Shelburne Iii
Yuequan Shen
Alan Sher
Edward Sherwood
Ethan Shevach
Meiqing Shi
Pei-Yong Shi
Chiaho Shih
Michael Shiloh
Hiroyuki Shimizu
Sunny Shin
Joanna Shisler
Charles Shoemaker
Sujan Shresta
Hong-Bing Shu
Masahiro Shuda
Lester Shulman
Stewart Shuman
Aleem Siddiqui
Inga Siden-Kiamos
Scott Sieg
Tim Siegel
M. Sloan Siegrist
Luis Sigal
Christina Sigurdson
Anita Sil
Robert Siliciano
Neal Silverman
Robert Silverman
Guido Silvestri
Olivier Silvie
Peter Simmonds
Lyle Simmons
Scott Simon
Raphael Simon
Viviana Simon
Carmen Simón-Mateo
Lone Simonsen
Anthony Sinai
Alison Sinclair
John Sinclair
Robert Sinden
Photini Sinnis
Christian Sinzger
Mark Siracusa
Anand Sitaram
Eric Skaar
Ann Skalka
Rebecca Skalsky
Christine Skerka
Pamela Skinner
Jacek Skowronski
Barton Slatko
James Slauch
Michel Slotman
Pamela Small
Geert Smant
James Smiley
Jolanda Smit
Gavin Smith
C. Jeffery Smith
Thomas Smith
Janet Smith
Deborah Smith
Duncan Smith
Phillip Smith
David Smith
Terry Smith
Sophie Smither
Megan Smithey
Sigrun Smola
Eric Snijder
Christopher Snyder
Francisco Sobrino
R. Sockett
Beate Sodeik
Donald Sodora
Evgeni Sokurenko
Isabel Sola
Dominique Soldati-Favre
Peter Solomon
Holger Sondermann
Kamoun Sophien
Maurizio Sorice
Tania Sorrell
Erin Sorrell
Claudio Soto
Hugo Soudeyns
Jayme Souza-Neto
Graça Soveral
Gregory Sowd
András Spaan
Gerald Spaeth
Stefania Spanò
Paul Spearman
Samuel Speck
Deborah Spector
Stephen Spector
Juliet Spencer
Vanessa Sperandio
Shiranee Sriskandan
Arun Srivastava
Raymond J. St. Leger
E.V. Stabb
Peter Staeheli
Simona Stäger
Jason Stajich
Christina Stallings
Leonidas Stamatatos
Thomas Stamminger
Richard Stanton
Glyn Stanway
Jack Stapleton
Vincent Starai
Michael Starnbach
Bärbel Stecher
Chad Steele

Olivia Steele-Mortimer
William Steinbach
Bettie Steinberg
Eike Steinmann
Ivo Steinmetz
Steffen Stenger
Robin Stephens
David Stephens
Ioannis Stergiopoulos
Paul Sternberg
Claus Sternberg
Silke Stertz
Alasdair Steven
Dennis Stevens
Mark Stevens
Mario Stevenson
Mary Stevenson
Adrie Steyn
Scott Stibitz
Benoit Stijlemans
James Stivers
Cheryl Stoddart
Heribert Stoiber
Henrik Stotz
Jens Stougaard
Jose Stoute
Jonathan Stoye
Klaus Strebel
Daniel Streblow
Thomas Strecker
Hendrik Streeck
Boris Striepen
Roland Strong
Lynda Stuart
Frank Stubenrauch
Eva Stukenbrock
Jason Stumhofer
Hendrik Stunnenberg
Lishan Su
Laura Su
Kanta Subbarao
Sriram Subramaniam
Agathe Subtil
Peter Sudbery
Andreas Suhrbier
William Sullivan
Christopher Sullivan
Paul Sumby
Joseph Sun
Jianjun Sun
Jie Sun
Qinmiao Sun
Ren Sun
Beicheng Sun
Frantisek Supek
Philip Supply
Bernd Sures
Michael Surrette
Mehul Suthar
Gerd Sutter
Fayyaz Sutterwala
Richard Sutton
Elena Suvorova
Nobuhiro Suzuki
Tetsuro Suzuki
Tetsuro Suzuki
Staffan Svard
Sankar Swaminathan
Michele Swanson
Joel Swanson
Ronald Swanstrom
W. Edward Swords
Gulam Syed
Christine Szymanski
Fabrizio Tagliavini
Andrew Tai
Ayato Takada
Emi Takashita
Toru Takimoto
Adel Talaat
Nicholas Talbot
Yunhao Tan
Hengli Tang
Qiyi Tang
Herbert Tanowitz
Yizhi Tao
Vera Tarakanova
Rick Tarleton
David Tarlinton
Ann Tate
Peter Tattersall
Norbert Tautz
Norbert Tautz
Martin Taylor
Terrie Taylor
Ronald Taylor
Matthew Taylor
Jesse Taylor
John Teijaro
Luis Teixeira
Antonio Teixeira
Glenn Telling
Timothy Tellinghuisen
Lesly Temesvari
Benjamin Tenoever
Ryohei Terauchi
Carolyn Teschke
Rita Tewari
Luc Teyton
David Thanassi
Ulrich Theopold
Dennis Thiele
Emmanuel Thomas
Vinai Thomas
Paul Thomas
Bart Thomma
David Thorley-Lawson
Scott Tibbetts
Leann Tilley
Jens Tilsner
John Tilton
Adrian Ting
Rabindra Tirouvanziam
Margaret Titus
David Tobin
Georgia Tomaras
Jan Tommassen
Keizo Tomonaga
Liang Tong
Qiong Tong
Hung Ton-That
Victor Torres
Jaume Torres
Jordi Torres
Domenico Tortorella
Zsolt Toth
Lhousseine Touqui
Greg Towers
Jonathan Towner
Jeffrey Townsend
Paula Traktman
Erin Tran
Lydie Trautmann
Ana Traven

John Treanor
Moritz Treeck
Katharine Trenholme
Steven Triezenberg
Mirko Trilling
Ralph Tripp
Billy Tsai
Anastasios Tsaousis
David Tscharke
Renée Tsolis
Moriya Tsuji
Allan Tsung
Paul Tudzynski
Bettina Tudzynski
Cagla Tukel
Terrence Tumpey
Elaine Tuomanen
Lynne Turnbull
Stephen Turner
Martin Turner
Joseph Turner
Joanne Turner
Rodney Tweten
Kenneth Tyler
Brett Tyler
Sukathida Ubol
Christel Uittenbogaart
Buddy Ullman
David Underhill
Meera Unnikrishnan
Derya Unutmaz
Jason Upton
Constantin Urban
Britta Urban
Kevin Urdahl
Peter Urwin
Edward Usherwood
Mart Ustav
Mart Ustav
Jude Uzonna
Akhil Vaidya
Sergei Vakulenko
Raphael Valdivia
Barbara Valent
Susana Valente
Richard Vallee
Guido van den Ackerveken
Philippe van den Steen
Peter van der Ley
Tom van der Poll
Giel van Dooren
Linda van Dyk
Peter Van Esse
Lucien van Keulen
Frank van Kuppeveld
Rene van Lier
Ronald van Rij
Jos van Strijp
Russell Vance
Scott Vande Pol
Alain Vanderplasschen
Ramakrishna Vankayalapati
Steven Varga
Dimitrios Vatakis
Ashley Vaughan
Andres Vazquez-Torres
Ronald Veazey
Jan-Willem Veening
Michael Veit
Patricia Veras
Jeanmarie Verchot-Lubicz
Subhash Verma
Kenneth Vernick
Gijs Versteeg
Laura Vesala
Frédéric Veyrier
Henri Vial
Cecile Viboud
Miguel Vicente-Manzanares
Silvia Vidal
Marco Vignuzzi
Rytas Vilgalys
Frederik Vilhardt
Karen Visick
Kumar Visvanathan
Jörg Vogel
Sebastian Voigt
Sarah Volkman
Waldemar Vollmer
Matthias von Herrath
Andreas von Tiedemann
Daniel Voth
Jovanka Voyich-Kane
Jatin Vyas
Ajai Vyas
Andreas Wack
Jonathan Wadsworth
Sun Wai
Gabriel Waksman
Matthew Waldor
Peter Walker
Mark Walker
Ross Waller
Robert Wallis
Sarah Walmsley
Pegine Walrad
Peter Walsh
Jue Wang
Xiaofeng Wang
Zonghua Wang
Bo Wang
Yufei Wang
Yan-Yi Wang
Yue Wang
Ping Wang
Guangshun Wang
Qing Jun Wang
Guo-Liang Wang
Yan-Jiang Wang
Robert Wang
Jonathan Warawa
Gary Ward
Andrew Ward
Brian Ward
Peter Ward
Honorine Ward
Carl Ware
Matthew Wargo
Digby Warner
Andrew Waters
Chris Waters
David Watkins
Joel Watts
Richard Webby
Friedemann Weber
Joanne Webster
Heiner Wedemeyer
Taiyun Wei
Christopher Weidenmaier
J. Brice Weinberg
Joel Weinstock
Janis Weis
Jeffrey Weiser
Daniela Weiskopf

Günter Weiss
Susan Weiss
Brian Weiss
David Weiss
Louis Weiss
Winfried Weissenhorn
Jonathan Weitzman
Matthew Weitzman
Sandra Weller
Susanne Wells
Dave Wemmer
Wolfgang Weninger
Marianne Wessling-Resnick
Michelle West
Hannah Wexler
Robert Wheeler
Stephen Whisson
Judith White
Bradley White
K. Andrew White
Richard Whitley
James Whitney
Gary Whittaker
Claude Wicker-Thomas
Reed Wickner
Matthew Wiebe
Matthew Wiebe
Lüder Wiebusch
Emmanuel Wiertz
Brian Wigdahl
Tom Wileman
Claus Wilke
Gavin Wilkinson
Gavin W.G. Wilkinson
Bryan Williams
David Williams
Kenneth Williams
Matthew Williams
Michael Williams
John Williams
Kim Williamson
Carolyn Williamson
Peter Williamson
Mark Wills
Emma Wilson
Sam Wilson
Mark Wilson
Brenda Wilson
R. Alan Wilson
Duncan Wilson
Ken Wilson
Ian Wilson
Alex Wilson
Anthony Wilson
Jeffrey Wilusz
Eckard Wimmer
Malcolm Winkler
Sebastian Winter
Elizabeth Winzeler
Charles Wira
Roger Wise
Thomas Wisniewski
Jeffrey Withey
Dominik Wodarz
Teddy John Wohlbold
Alan Wolfe
Thorsten Wolff
Matthew Wolfgang
Adrian Wolstenholme
Andrea Woltman
Christiane Wolz
Craig Woodworth
Matthew Woolard
Floyd Wormley, Jr.
Michael Worobey
Daniel Wozniak
Jens Wrammert
Elizabeth Wright
Min Wu
Louisa Wu
Zhijian Wu
Ting-Ting Wu
Jennifer Wuerth
Betty Wu-Hsieh
Richard Wyatt
David Wyles
Thomas Wynn
Zanxian Xia
Hui Xiao
Xiufang Xin
Zhou Xing
Chaoyang Xue
Timothy Yahr
Masahiro Yamashita
Jie Yan
Nan Yan
X. Frank Yang
Xi Yang
Ruifu Yang
George Yap
Robert Yarchoan
Jun-Ichirou Yasunaga
Maria Yazdanbakhsh
Yihong Ye
Jonathan Yewdell
Minkyung Yi
Fitnat Yildiz
Timothy Yoshino
Janet Yother
Jianxin You
Dahui You
Kevin Young
Ryland Young
Vincent Young
Lawrence Young
Benjamin Youngblood
Jacob Yount
Xiao-Fang Yu
Xu Yu
Jae-Hyuk Yu
Peihua Yuan
Yan Yuan
Kwok-Yung Yuen
Sung-Hwan Yun
Andrew Yurochko
Mark Zabel
Olga Zaborina
Jerome Zack
Dietmar Zaiss
Allan Zajac
Xingxing Zang
Gianluigi Zanusso
Colby Zaph
Fidel Zavala
Francisco Zerbini
Kornelius Zeth
Bing Zhai
Lian-Hui Zhang
Pei Zhang
Zhang Zhang
Junjie Zhang
Xuming Zhang
Luwen Zhang
Xiuren Zhang

Kai Zhang
Jincun Zhao
Xiao-Dong Zhao
Yong-Hui Zheng
Guangming Zhong
Z. Hong Zhou
Rui Zhou
Xueping Zhou
Guan Zhu
Fanxiu Zhu
Joseph Ziegelbauer
Daniel Zilberman
Jochen Zimmer
Peter Zipfel
Nicole Zitzmann
Elina Zuniga
Jian Zuo
Chiara Zurzolo
Larry Zweibel
Michael Zwick
